# Association between Anatomical Features of Petrotympanic Fissure and Tinnitus in Patients with Temporomandibular Joint Disorder Using CBCT Imaging: An Exploratory Study

**DOI:** 10.1155/2020/1202751

**Published:** 2020-07-24

**Authors:** Edward Kijak, Agnieszka J. Szczepek, Jerzy Margielewicz

**Affiliations:** ^1^Department of Prosthetic Dentistry, Faculty of Medicine and Dentistry, Pomeranian Medical University, Rybacka 1, 70-204 Szczecin, Poland; ^2^Department of Prosthetic Dentistry, Faculty of Medicine and Dentistry, Wroclaw Medical University, Ludwika Pasteura 1, 50-367 Wrocław, Poland; ^3^Department of Otorhinolaryngology‐Head and Neck Surgery, Charité–Universitätsmedizin Berlin, Corporate Member of Freie Universität Berlin, Humboldt-Universität zu Berlin, and Berlin Institute of Health, 10117 Berlin, Germany; ^4^Silesian University of Technology, Faculty of Transport and Aviation Engineering, Krasińskiego 8, Katowice 40-019, Poland

## Abstract

Mandible displacement is known to correlate with otological conditions such as pain in the ear canal, hearing loss, or tinnitus. The present work aimed to determine the association between the displacement of the condyle in a temporomandibular joint, the structure and position of the petrotympanic fissure (PTF), and comorbid tinnitus in patients affected by temporomandibular joint and muscle disorder (TMD). We enrolled 331 subjects with TMD (268 women and 63 men). The average age of women was 40.8 ± 16.8 years (range 13–88), whereas the average age of the examined men was 38 ± 14 years (range 13–74). We performed imaging studies of the facial part of the skull in the sagittal plane using a volumetric imaging method and a large imaging field (FOV) of 17 cm × 23 cm. The habitual position of the mandible was determined and used as a reference. Based on the imaging results, we developed a classification for the topography and the structure of the petrotympanic fissure. Thirty-three TMD patients (about 10% of the sample) reported having tinnitus. These patients had PTF configurations characterized by a rear (36.59%) or intracranial-cranial (63.41%) condylar displacement of the temporomandibular joint. Our findings imply that the TMJ- and tinnitus-positive group of patients possibly represents a distinct phenotype of tinnitus. We concluded that for such patients, the therapeutic approach for tinnitus should include TMD treatment.

## 1. Introduction

Tinnitus is a subjective perception of sound without an external acoustic signal occurring due to inappropriate activation of the auditory cortex. Tinnitus may be perceived as unilateral or bilateral phantom sound and be either continuous or intermittent symptom [1] of various conditions, including the malfunction of auditory periphery [2]. Activation of the auditory cortex of tinnitus patients in silence has been documented during imaging studies using positron emission tomography (PET) or functional magnetic resonance imaging (fMRI) [3]. Tinnitus may be a symptom of many conditions, including presbyacusis, otosclerosis, chronic otitis media, ototoxicity, labyrinthitis, noise-induced hearing loss, and congenital disorders. Besides, diseases that directly or indirectly affect neurons of the auditory pathway (multiple sclerosis, vestibular schwannomas, meningiomas, stroke, intracranial hemorrhage, and head injuries) may also associate with tinnitus. Tinnitus patients have often compromised hearing thresholds and speech perception and generally have a lower health-related quality of life [4].

Furthermore, comorbid conditions such as insomnia, anxiety, difficulties with concentrating, or negative thinking may induce a vicious circle, in which tinnitus causes progression of psychological problems, and they, in turn, induce progression of tinnitus [5, 6]. Approximately 15–20% of the adult population is affected by tinnitus, making tinnitus a severe socioeconomic problem [7]. Only in Europe, more than 70 million people report having tinnitus [8]. Psychosocial and emotional stresses play a particular role in the onset and chronification of tinnitus [9].

Emotional stress is one of the causes of masticatory muscle disorders, characterizing the temporomandibular disorders (TMD). The impact of psychological factors on the development of TMD is well documented. It can exemplarily be demonstrated by the high incidence of TMD in patients with a major depressive disorder [10–12]. Temporomandibular disorders are the third global dental problem after caries and diseases of the periodontium; however, they may be challenging to diagnose, especially in earlier stages. The leading causes of TMD include tendomyopathy and degenerative disease of the temporomandibular joint, among others [13]. TMD affects over 10% of the world population, and about 40% of patients with TMJ experience pain, whereas their age at diagnosis is steadily declining [14]. Epidemiological studies demonstrated that as much as about 75–90% of the Western population suffers from functional disorders of the stomatognathic system, with female gender skew [15–17]. Pain, jaw movement restriction, and acoustic effects, including popping and clicking in the joints, are considered the classic triad of symptoms of TMD [18]. In patients with the severe form of TMD, the main symptoms may be accompanied by a limited range of mandible movement, headache, or pain in the neck. Headaches are a common problem for participants with painful TMD [19]. Less frequent are the otolaryngological symptoms such as ear pain, hearing loss, sudden hearing loss, a feeling of fullness in the ear, sore throat, difficulty in swallowing, or tinnitus [20].

TMD-related tinnitus is considered to represent the somatosensory type of tinnitus [21, 22] and is diagnosed in 10–15% of the population with TMD [23]. Early research on TMD and comorbid tinnitus initiated by Costen described clinical cases of different otological conditions associated with a loss of teeth in the distal part of the dental arch, posterior support, or loss of prosthetic vertical dimension [24]. Costen proposed the mechanical compression of the auriculotemporal nerve to be caused by the dorsocranial condylar displacement, which in turn causes pain and various types of otalgia. There are as many supporters as opponents of Costen's theory. The condylar displacement can also induce compression of the synovial petrotympanic fissure, which directly communicates with the middle ear. All anatomical structures near synovial petrotympanic fissure, such as chorda tympani, branch of the facial nerve (VII), anterior tympanic artery and vein, anterior process, and anterior ligament of the malleus, could also be affected and therefore lead to otalgia. However, the reasons for tinnitus that only occasionally accompanies the TMD remain unclear.

In this research, we attempted to explain the link between the occurrence of tinnitus and the temporomandibular joint topography in TMD patients and to determine TMD-related factors that might be predisposing for tinnitus. To obtain the anatomical information, we used the X-ray imaging–CT volumetric analysis. The presence or absence of tinnitus was determined as an answer to an open question. We focused our analysis on the relationship between the structures of petrotympanic fissure (PTF), the condylar displacement of the temporomandibular joints in patients with TMD, and the presence of tinnitus.

## 2. Materials and Methods

The Ethics Committee approved this retrospective study of the Pomeranian Medical University, Szczecin, Poland (approval number KB-0012/30/13) and waived the need for informed consent. The study was conducted according to the principles in the Declaration of Helsinki.

The study group counted 331 patients who reported to the Department of Prosthodontics (Pomeranian Medical University in Szczecin, Poland) due to temporomandibular joint disorder in the years 2016–2018. Of the 331 subjects, 268 were women (age range between 13 and 88 years; average age 40.84 ± 16.71) and 63 were men (age range between 13 and 74 years; average age 38.5 ± 14.59 years). Women accounted for 80.97% of the study population and men for 19.03%, which is in agreement with earlier epidemiological observations of TMJ incidence. Although one patient in our cohort, who was below 18 years of age, was initially included in the study, this person was excluded from the data analysis because of the still ongoing process or cranial development—fissures are fulfilled with fibrous structure.

The selection of patients qualified for further analyses was based on the II RDC/TMD axis of diagnostic criteria for temporomandibular disorders (RDC/TMD) introduced by Dworkin and LeResche in 1992 [25]. The inclusion process was based on the existing medical records of previously diagnosed patients according to the accepted standards developed by the International Consortium RDC/TMD [26]. The inclusion criteria comprised medical records about existing or past temporalmandibular joint disorders consistent with group II of joint disorders. The selection also included the presence of otolaryngological symptoms, such as ear pain, hearing loss, sudden hearing loss, a feeling of fullness in the ear, or tinnitus. Since the selection was made based on medical documentation, the psychological status of the subjects was not assessed according to RDC/TMD Axis II diagnoses. Consequently, the study group consisted of persons who meet the criteria for groups IIa and IIb of the said classification. All participants underwent a thorough assessment in accordance with the RDC/TMD guidelines to receive Axis II diagnoses based on the official Polish adaptation of the RDC/TMD [27].

The exclusion criteria comprised previous mechanical injuries to the mandibular joint area: fractures, dislocations, former or present ear diseases (including infections), and hearing impairment. Another exclusion criterion was treatment of the dysfunction with occlusal splints and subjects without stable maxillamandibular relation.

The second eligibility condition was a previously performed CBCT imaging test. Only the examinations that were performed using the same technique and focusing on the same imaging field were considered for analysis. An area of view (17 cm × 23 cm) was obtained during craniofacial volumetric tomography (FOV), using a scanner for i-CAT Next Generation (ISI) cranial tomography (Hatfield, PA, USA) and software version 1.9.3.13. The applied exposure protocols were the default and manufacturer-recommended protocol (exposure settings: 90 kV, 5 mA, and 16.0 s). The head position with the Frankfurt plane paralleled, and the midsagittal plane was perpendicular in relation to the floor of the mouth. Patients were informed to sit still during exposure. Multiplanar data were reconstructed with a pixel size of 0.25 mm.

A total of 662 scans of temporomandibular joint (left and right for each of 331 patients) were analyzed in the sagittal plane in reference position, musculoskeletal position, or maximal intecuspidation. Habitual position is not repetitive and, in TMD patients, is often the result of pathological habit. It is impossible to take it under consideration. Such a stable occlusal relationship faithfully reflects the actual position of the condylar process concerning the surrounding anatomical structures.

### 2.1. Image Analyses

Based on the CT images, we performed a detailed analysis of petrotympanic fissure topography. Because the structure of craniofacial bone is of high diversity and there is a lack of existing evaluation criteria, we have developed our classification. It aimed at categorizing the craniofacial bone topography.

The analyses of CBCT images were performed by one person (the first author of the study), well experienced in evaluating this type of imaging. The evaluation steps, methodology, and reproducibility were established in collaboration with a professional radiologist. The analysis was performed using a personal computer equipped with an Intel(R) Core(TM) i7 4720HQ CPU and a 64 bit Windows version 8.1 operating system.

**Figure 1:**
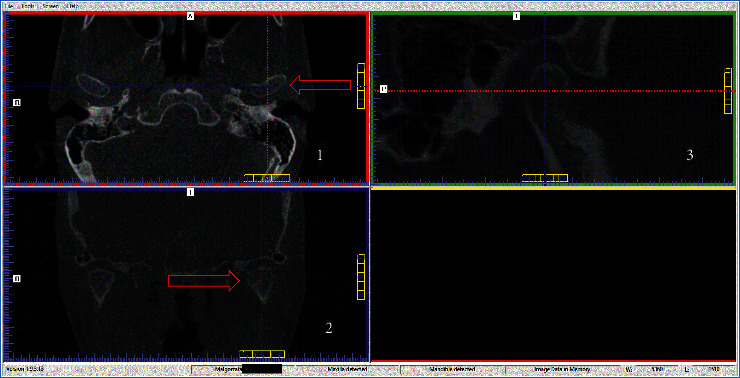
Steps of the petrotympanic fissure assessment of in the CBCT image.

CBCT analysis was initiated by identifying the condylar process in the frontal plane window. After setting the intersection axis of the planes, the center of the condyle of mandible was determined in two horizontal (Figure 1, panel 1) and frontal (Figure 1, panel 2) planes. The enlarged image of the petrotympanic fissure was then analyzed (in the sagittal plane) in terms of the parameters tested (Figure 1, panel 3).

**Figure 2:**
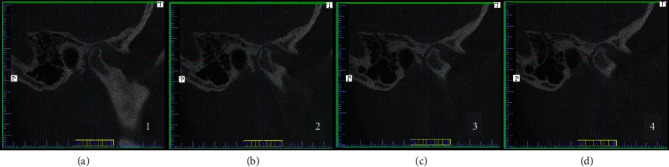
Verification of reproducibility of analyses. (a–d) CBCT images of various patients from the analyzed cohort.

Next, the individual scans depicting the petrotympanic fissure were analyzed without changing the position of the planes positioned at the beginning of the analyzes. In this way, the reproducibility of analyzes in the remaining cases was achieved (Figure 2.)

**Figure 4:**
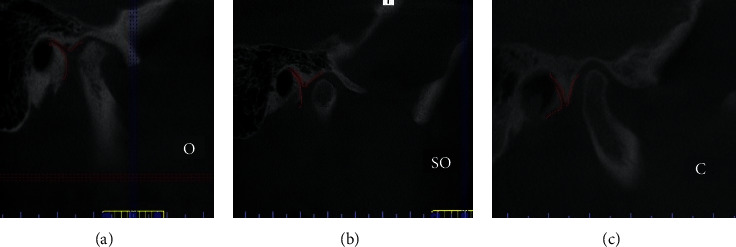
Classification of the entrance shape of a petrotympanic fissure: (a) open (O), (b) semiopen (SO), and (c) closed (C).

Further analysis revealed that the entrance to the petrotympanic fissure could also be categorized into three distinct classes: open (O), semiopen (SO), and closed (C) (Figure 4).

Thus, the following combinations of the positioning and entrance shape are possible for petrotympanic fissure: L-O; L-SO; L-CR; M-O; M-SO; CR-M; H-O; H-SO; and H-CR.

**Figure 5:**
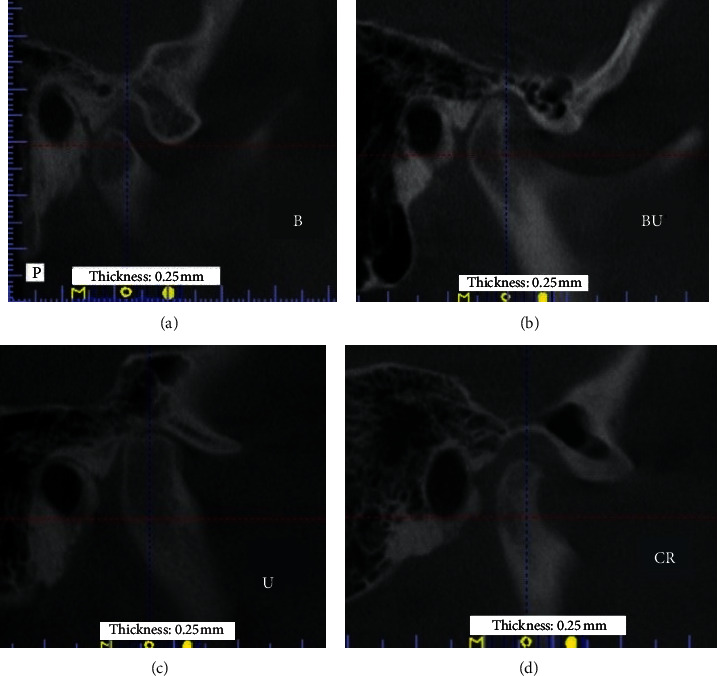
Classification of the condylar position: (a) rear (B); (b) top-rear (BU); (c) intracranial-cranial (U), and (d) unchanged with no evidence of displacement (CR).

To address Costen's theory assuming that the otological symptoms are related to the dorsocranial condylar displacement, the analysis of the topographical elements of the joint in the sagittal plane was performed, and four types of condylar position were identified (Figure 5):

- Rear (B)
- Top-rear (BU)
- Intracranial-cranial (U)
- Unchanged with no evidence of displacement (CR)

The positioning and entrance shape of petrotympanic fissure is closely related to the length and width of the PTF channel. However, we have not used these parameters in our analyses for two reasons: first, it was because of already identified relations, and second, it was because both parameters are resulting from the positioning and shape (type) of PTE entrance.

### 2.2. Statistical Analysis

For the statistical calculations, we used Mathematica 10.4 software (Wolfram Research, Oxfordshire, United Kingdom). For all statistical analyses, the significance level (*p*) was set on 0.05. Based on the Shapiro–Wilk statistical test, the data obtained from the test group of women were not consistent with normal distribution because of the random variable representing the age. This conclusion was drawn from the fact that the probability value of the Shapiro–Wilk test (*p*) was ≤0.0001. In the group of male patients, the probability value of the Shapiro–Wilk test was *p* ≤ 0.0002, which also supports the noncompliance with the normal distribution. In terms of age, the group of men and women did not differ significantly (the value of the nonparametric Mann–Whitney test was *p*=0.066), allowing analysis of both genders together.

The analysis was continued with data, in which the skeletal structure of the face was presented in quantitative form. Before the correlation analyses, a proportion of individual factors in the analyzed group was determined. Statistical analysis (Person's correlation) *χ*^2^ demonstrated that the age of women in our sample does not differ significantly from that of men (*p* value = 0.0566). Therefore, the two groups could be compared.

## 3. Results

**Figure 6:**
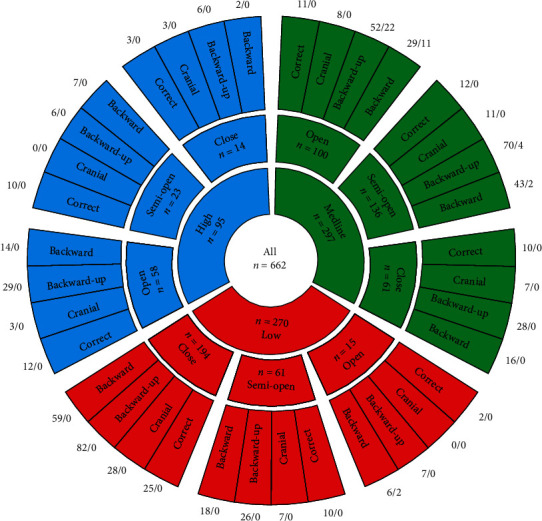
Permutations (without repetition) of parameters characterizing the skeletal and articular structures of petrotympanic fissure and the corresponding numbers of tinnitus occurrences.

First, we determined the frequency of symptom occurrence in our sample. Due to a large number of parameters analyzed and to visualize the results, we developed a customized presentation of results. Three rings represent the main parameters: the center ring reflects the frequency of a given position of the petrotympanic fissure, whereas the middle ring contains information about the frequency of a given entrance to the petrotympanic fissure. The outside ring includes information on the topography of the temporomandibular joints in the sagittal plane. The numerical values (represented by two numbers) defining individual configurations of condylar positions are placed outside the circle. The first number denotes the frequency of occurrences of a given setting, whereas the second number represents the number of tinnitus cases (Figure 6).

**Figure 7:**
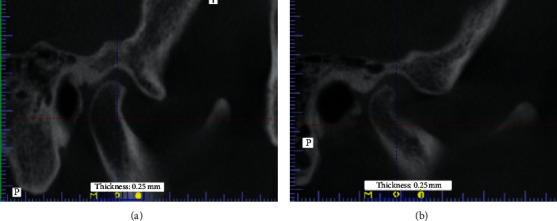
CBCT image of the topography of temporomandibular joint of the patients with tinnitus during the course of dysfunction: (a) 58-year-old female patient; (b) 68-year-old female patient.

Figure 7(a) demonstrates an example of location and type of entrance to the petrotympanic fissure and top-rear, while Figure 7(b) illustrates the rear displacement (Figure 7(b)) in patients with TMJ and tinnitus. The CBCT image shows the apparent condylar displacement of a significant degree, virtually closing the entrance to the petrotympanic fissure with radiological signs of bone destruction.

Tinnitus was often reported by patients with rear (36.59%) or intracranial cranial (63.41%) condylar displacement of the temporomandibular joint (*p* < 0.05). According to Costen, such condylar displacement is necessary (but not sufficient according to our findings) for the occurrence of tinnitus. The shape and the position of petrotympanic fissure significantly correlate with the presence of tinnitus. In detail, the presence of tinnitus associates with open (85%) or semiopen (14.63%) form of entrance to petrotympanic fissure and with its midline (95.12%) or low position (4.88%).

**Figure 8:**
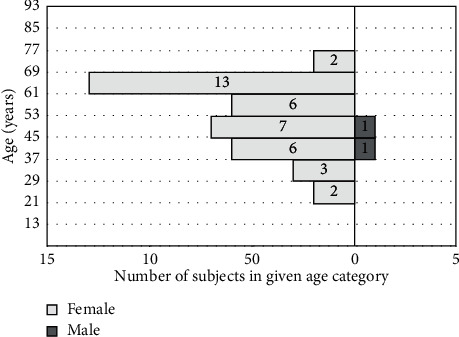
Association between subject age and the perception of tinnitus.

In the analyzed group of participants, 33 subjects (9.97%) reported tinnitus, of which 2.42% had bilateral tinnitus. The prevalence of tinnitus was higher in women (93.75%) than in men (6.25%) (Figure 8), stressing the gender-related aspect in TMJ/tinnitus patients.

Table 1 contains the results of Fisher-Pitman permutation analyses used to test the null hypothesis about the correlation between the position plus type of entrance to petrotympanic fissure and the incidence of tinnitus. The reason for including only women was that only two men reported tinnitus. Both of these men had midline position “M” of the petrotympanic fissure entrance. One man had an open type “O,” whereas the second had a semiopen “SO” type of the entrance shape. In both cases, the mandible was in rear position “B.”

Probability was calculated using a chi-squared test, with the *p* level of significance set for 0.05. The resulting values indicate a lack of statistically significant differences in the occurrence of tinnitus when the same configuration of petrotympanic fissure in the left and right pond was present.

Our analysis provided additional information regarding the correlation between the age of patients with TMJ and the occurrence of tinnitus. The average age of patients with TMJ and tinnitus was above 40, suggesting that both symptoms occur predominantly in mature and older patients (Figure 8).

## 4. Discussion

The present work demonstrates that tinnitus is reported by 10% of 331 subjects with TMJ, corroborating the findings of other researchers who determined the prevalence of tinnitus in subjects with TMJ being 7.28% [28], 10% [29], or 15–20% [7]. However, Manfredini et al. reported the prevalence of tinnitus in patients with TMJ as high as 30.4%, which may be due to a difference in inclusion criteria or collection of information about tinnitus [30]. The novel finding of the present work is that the occurrence of tinnitus associates with open (85%) or semiopen (14.63%) form of entrance to petrotympanic fissure and with its midline (95.12%) or low position (4.88%).

Over 85 years ago, Costen introduced the medical term “temporomandibular joint dysfunction” [24]. Costen described the syndrome based on eleven cases as a triad of temporomandibular joint complaints, headaches, and sensory disturbances in the area of the oral cavity. Costen suggested that the approximation of the lower jaw to the upper jawbone associated with a loss of posterior teeth may be the cause of TMJ [24]. Over the years, a lot of disagreement over Costen's theory has accumulated. Peroz pointed out that the functional disorders of the masticatory system may be accompanied by ear pain (37%) and tinnitus (3.7%) [31]. Peroz also suggested that otological symptoms with negative otoscopy might indicate TMJ. Keersmaekers et al. reported otological symptoms in 42% of patients with TMJ [32]. Using magnetic resonance imaging (MRI), Seedorf and Judah demonstrated that the prevalence of ear pain, as a primary symptom in TMJ, could reach 7.1% [33]. Tuz et al. recognized ear pain as the most common symptom in patients with TMJ with an incidence of up to 63% [34].

To date, there is a lack of evidence directly supporting the correlation suggested by Costen. Some researchers consider TMJ to be a multifactorial and multisymptomatic disease, with psychosocial elements [35]. Some others refer to the etiology of TMJ as idiopathic [36]. However, the anatomic features of the ear, one of the most specialized sensory organs in people [37] and the temporomandibular joint, seem to support Costen's theory. The articular disk of the temporomandibular joint is located in the temporal bone only 1 to 2 mm from the external auditory meatus. The proximity between the hearing and balance organs and the temporomandibular joint determines their common vascularization and innervation by the cranial nerve V (trigeminal nerve). Petrotympanic fissure provides direct contact between the auricular cavity and the middle ear. Often divided into petrotympanic or petrosquamous, the petrotympanic fissure may be used by infectious pathogens for a bidirectional transmission [38]. Moreover, the displacement of anatomical structures in the temporomandibular joint can cause a variety of symptoms associated with otological dysfunctions. One of the multiple theories attempting to explain the association between otological symptoms and TMD assumes that TMD results are either from excessive mechanical pressure on the discomalleolar ligament or from direct strain on the auriculotemporal nerve [39, 40]. It is also believed that the increased muscle tension of the jaw observed in patients with TMJ might result in a significant increase of the pressure on the temporomandibular joints and, thus, overloaded the surrounding tissues and muscles [38]. Although in our sample, all subjects had arthritis, the presence of increased muscle tension cannot be excluded. Muscle tension as a response to psychosocial stressors occurs in all patients with TMJ.

Interestingly, many reports indicate that TMD patients complain more often of tinnitus than of jaw problems [41]. The structures within the petrotympanic fissure (e.g., an anterior tympanic branch of the internal maxillary artery) could likely undergo mechanical stimulation due to the changes in the tension of maxillary muscles [34]. That, in turn, could lead to changes in cochlear microcirculation, such as hypoxia or even ischemia, and both of them are known to significantly affect the cochlear function [42, 43] by inducing degenerative processes, which in some patients could be responsible for the generation of tinnitus [44].

The temporomandibular joints are characterized by high adaptability and regeneration. However, after exceeding their regenerative capacity, the destructive changes that follow may be irreversible. Masticatory functional disorders occur in every younger patient. We noted that the average age of TMD patients with tinnitus was about 40 years, and our younger patients have not reported tinnitus. It is not in agreement with audiological research, which found that tinnitus occurs less frequently in patients younger than 54 years. Lockwood observed that in a general population over 55, about 30% complain of tinnitus [45, 46].

Çakur and colleagues classified PTF into three types: type 1 (wide tubular formation), type 2 (double conical structure), and type 3 (single conical structure) [47]. The same group has studied the correlation between the anatomic features of the petrotympanic fissure and the occurrence of tinnitus. The authors conducted CBCT imaging in 100 TMD patients, of whom 50% reported tinnitus and concluded that short fissure with wide entrance might predispose TMD patients to tinnitus (Çakur and Yasa, 2016) [48]. However, there are significant differences between the study of Çakur and ours. First, we analyzed the localization and the type of entry to the petrotympanic fissure and found them to be closely related to the length of PTF. The fissures belonging to the closed type are long and narrow. Therefore, we reasoned that the development of microcirculation conditions in the fissure vessels would be unlikely. Our study corroborates the notion about the close correlation between the location, the shape of the entrance to the petrotympanic fissure, and the rear condylar displacement. Interestingly, 95% of subjects with such a configuration reported tinnitus. This is the first report on the positive correlation between anatomical features of the petrotympanic fissure and the occurrence of tinnitus. Second, our sample was significantly larger (330 patients in our study vs. 100 patients in Çakur study) and differed regarding gender distribution and occurrence of tinnitus. Last, all our patients were referred to our unit because of group II-disorders of synovial character. In contrast, Çakur et al. used the presence of systemic diseases as one of the exclusion criteria.

There was no correlation between gender and the incidence of tinnitus in our sample. However, there was unequal gender distribution (about 80% women and 20% men), which was in agreement with epidemiological data indicating that TMD is diagnosed 3-4 times more often in women than in men. Miekle and Griest (1989) found that the majority of men suffering from tinnitus were exposed to noise [49]. According to Baguley and McFerran, the cause of tinnitus cannot be explicitly determined in about 67% of all tinnitus cases, which could reflect the changes in central auditory processing [50]. Disc dislocation may also play an important role in tinnitus generation. In agreement with that, Costa et al. confirmed that the more massive the disc displacement, the higher the incidence of effusion [51]. The presence of intra-articular effusion may also play a significant role in the generation of tinnitus.

The major pitfall of our study is a small sample of the TMD- and tinnitus-positive subjects. Future studies with a larger number of such patients should add statistical weight to our present observations. Another drawback of our research is a lack of audiological data generated by pure tone audiometry, the speech comprehension test, tympanometry, tinnitus matching, the minimum masking level, or the loudness discomfort level. Furthermore, the use of translated and validated psychometric instruments measuring tinnitus-induced distress, such as the Tinnitus Functional Index or Tinnitus Handicap Inventory, would be of great advantage [52]. Last, having a control group without TMJ could add insides to this type of research.

The present study aimed to elucidate the causative association between TMD-associated pathologies and tinnitus. Presented research does not support the existence of Costen syndrome. Based on the results, we conclude that the location and type of petrotympanic fissure may be a predisposing factor for tinnitus, especially in patients with TMD. Moreover, our research suggests that the nature of condylar displacement may be essential for tinnitus induction. Further studies should be conducted to extend the presented findings and to contribute to tinnitus phenotyping and the development of effective causative therapy for patients with tinnitus and TMD.

## 5. Conclusions

1. This study documents the association between tinnitus and TMJ
2. The location and type petrotympanic fissure may be a predisposing factor for tinnitus, especially in patients with TMJ
3. The type of condylar displacement of the temporomandibular joint may be essential for tinnitus induction

## Figures and Tables

**Figure 3:**
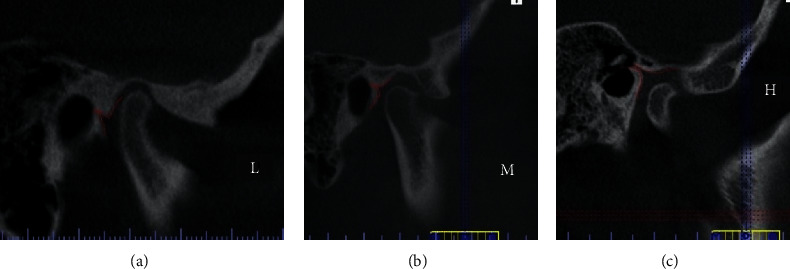
Example of three types of petrotympanic fissure positioning: (a) low-L, (b) midline-M, and (c) high-H (section in the sagittal plane). We distinguished three types of PTF position, marked with the appropriate symbols: low-L, midline-M, and high-H (Figure 3).

**Table 1:** Incidence of tinnitus in female subjects (*p* < 0.05).

Position	L, *n* = 2		M, *n* = 17 (94.9%)							
Type of entrance	Opened (5.1%)		Opened, *n* = 32 (82.1%)				Semiopened, *n* = 5 (12.8%)			
Condylar position	B		B		BU		B		BU	

Side	Right	Left	Right	Left	Right	Left	Right	Left	Right	Left
The number of tinnitus-positive cases (%)	0 (0)	2 (5.1)	6 (15.4)	4 (10.3)	10 (25.6)	12 (30.8)	1 (2.6)	0 (0)	3 (7.7)	1 (2.6)
*p*	0.234		0.424		0.509		0.317		0.316	

## Data Availability

The data used to support the findings of this study are available from the corresponding author upon request.
